# Exosomal MicroRNA-374b-5p From Tubular Epithelial Cells Promoted M1 Macrophages Activation and Worsened Renal Ischemia/Reperfusion Injury

**DOI:** 10.3389/fcell.2020.587693

**Published:** 2020-11-26

**Authors:** Chenguang Ding, Jin Zheng, Bo Wang, Yang Li, Heli Xiang, Meng Dou, Yuxi Qiao, Puxun Tian, Xiaoming Ding, Wujun Xue

**Affiliations:** ^1^Department of Kidney Transplantation, Nephropathy Hospital, The First Affiliated Hospital, Xi’an Jiaotong University, Xi’an, China; ^2^Institute of Organ Transplantation, The First Affiliated Hospital, Xi’an Jiaotong University, Xi’an, China; ^3^Department of Materials Science and Engineering, Monash University, Melbourne, VIC, Australia

**Keywords:** renal ischemia/reperfusion injury, tubular epithelial cells, macrophage, exosomes, miR-374b-5p

## Abstract

Tubular epithelial cells (TECs) represent the primary site of renal ischemia/reperfusion injury (RIRI). However, whether the damage of TECs could drive the initiation of inflammation was unclear. Here we investigated the role of the TECs and macrophages during RIRI. Increased expression of inflammation response and activated M1 macrophage were determined in the mice model of RIRI. Moreover, we demonstrated global miRNA expression profiling of renal exosomes, and miR-374b-5p was most upregulated in these exosomes *in vivo*. Inhibition of miR-374b-5p in the mice upon RIR operation would alleviate the kidney injury via decreasing the production of proinflammatory cytokines and suppressing the macrophage activation. Similar results were also identified in the hypoxia-induced cell model where exosomal miR-374b-5p was dramatically upregulated. Uptake of exosomes derived from the hypoxic TECs by macrophages would trigger M1 polarization via transferring miR-374b-5p. Besides, we confirmed that miR-374b-5p could directly bind to *Socs1* using a dual-luciferase reporter assay. Notably, when we injected the miR-374b-5p-enriched exosomes into mice, a high-level inflammatory response and M1 macrophage activation were performed. Our studies demonstrated that exosomal miR-374b-5p played an essential role in the communication between injured TECs and macrophages, resulting in the M1 macrophage activation during RIRI. The blockage of the release of such exosomes may serve as a new therapeutic strategy for RIRI.

## Introduction

Ischemia and subsequent reperfusion injury usually occur when the blood flow is blocked during surgeries or severe diseases. For the kidney, cardiac dysregulations and surgical interventions, such as kidney transplantation or nephron-sparing surgery, are the leading causes of RIRI (Venkatachalam et al., 2015; Skube et al., 2018). This lesion results in AKI and presents the fundamental problems and even contributes to a high morbidity and mortality rate (Chawla et al., 2014). For this purpose, treatments are studied to reduce the high morbidity and mortality, as well as recover renal function, while the optimal therapeutic approach is still lacking (Zuk and Bonventre, 2016).

Emerging data have reported that RIR regulated the fate of kidney and associated cells via triggering a long-term inflammation (Brodsky et al., 2002). In such settings, compelling evidence indicates that TECs, which are most susceptible to hypoxia, may not maintain the high metabolic rate to fulfill their functions and undergo death when the long-term inflammation is triggered (Kemmner et al., 2019). On the other hand, however, the hypoxic TECs can also facilitate the inflammation response by producing many inflammatory mediators such as MCP-1, TNF-α, and IL-1β, acting as driving factors in kidney diseases (Meldrum et al., 2003; Siegel and Shah, 2003; Lv et al., 2018; Wong et al., 2018). Nevertheless, it remains unclear how damaged TECs are involved in renal inflammatory reaction. One of the possible mechanisms could be the crosstalk between TECs and macrophages. It has been described that many molecules, including signal transducer and activator of transcription 1, Toll-like receptor 4, colony-stimulating factor 1, and MCP-1 have been increased activated under hypoxic condition, leading to upregulated macrophage infiltration and recruitment in kidneys (Wu et al., 2007; Menke et al., 2009; Ha et al., 2018; Lv et al., 2018). Subsequently, in response to tissue injury, macrophages ranged from the anti-inflammatory phenotype of M2 cells to the proinflammatory phenotype of M1 cells and activated and persisted in the progression of RIRI (Tang et al., 2019). Thus, the mechanism of how the TECs transfect these inflammatory signals into macrophage during RIRI still needs to be revealed.

To date, exosomes are generated by almost all cell types and are considered as the bridge among different cells (van Niel et al., 2018). They harbor many kinds of constituents, including nucleic acids, proteins, and metabolites, which are shed from the original cells. Recent studies demonstrated that various cells, such as epithelial cells, adipocyte, and endothelial cells, were identified to secrete more exosomes with hypoxia stimulation (Borges et al., 2013; Sano et al., 2014; Wong et al., 2018). Moreover, compared with the normoxic exosomes, hypoxia stimulation could also alter the contents of exosomes (Duan et al., 2019). Ikeda etal. found the production of urinary exosomal aquaporin-1 and aquaporin-2 was reduced when the rat subjected to RIR induced AKI, indicating they might be the potential biomarkers for RIRI detection (Asvapromtada et al., 2018). Besides, exosomes offer a window to switch the recipient’s phenotype and functions (Tkach and Thery, 2016; Skube et al., 2018). Some groups figured out the exosomes derived from mesenchymal stem cells exhibited promising protect effect on kidneys against RIRI, which could be used as a useful supplement for RIRI treatment (Lin et al., 2016; Li et al., 2019; Wang et al., 2019). Lin etal. also demonstrated an increased expression of transcriptional repressor activating transcription factor 3 in exosomes from epithelial cells and suppressed macrophage migration (Chen H. H. et al., 2014). In addition to these proteins, many studies also reported that exosomal miRNAs are involved in the communication between epithelial cells and macrophages. For instance, miR-19b-3p had been transferred into macrophages from TECs and displayed a harmful effect on kidney injury (Lv et al., 2020). Moreover, exosomal miR-23a could also participate in RIRI formation through activating M1 macrophages (Li et al., 2019). However, because of the complexity of cellular events, the role of exosomal miRNAs remains unclear. Accordingly, in this study, we aimed to determine the profile and mechanisms of injured TECs derived exosomes in promoting M1 macrophage activation and worsen the RIR-induced kidney injury.

## Materials and Methods

### Animals and Treatment

Male C57BL/6 mice (6–8 weeks, 22–25 g) were purchased from Charles River Labs (Beijing, China) and maintained on a standard diet and water provided *ad libitum*. The RIRI model was established as a previous study (Mehrotra et al., 2019). In brief, after anesthetizing with sodium pentobarbital (50 mg/kg, i.p.), the renal pedicle of mice was separated and clamped to induce bilateral renal ischemia. Then the clamp was removed 30 min later to allow reperfusion. Finally, the abdominal wall was sutured, and the mice were allowed to recover. Control mice underwent the same surgical procedures without clamping. Mice were sacrificed on days 1 and 3 after RIRI, and the kidneys and serum were harvested for further study. For intraparenchymal injection, the mice were anesthetized, and the left kidney was exposed; 20 μg exosomes [in 20 μL phosphate-buffered saline (PBS)] or equal-volume PBS was randomly injected into five sites of the left kidney via a 50-gauge needle (Li et al., 2019). For the *in vivo* transfection, 50 μg miR-374b-5p inhibitor or related control (inhibitor-NC) was mixed with the *in vivo* jetPEI reagent (Polyplustransfection SA, United States) following the manufacturer’s protocol and then transfected into mice kidney via tail vein injection 1 day before RIR operation. One day after RIR operation, the serum and kidneys were harvested.

### Serum Creatinine and Blood Urea Nitrogen

According to the manufacturer’s protocol, the serum was collected to detect the concentration of BUN by a kit (Stanbio Laboratory, United States) according to the manufacturer’s protocol. The level of SCr was assessed by the Jaffe reaction–based kit (Stanbio Laboratory, United States).

### Quantitative Real-Time Polymerase Chain Reaction

Total RNA from cells, exosomes, or kidney tissues were extracted using TRIzol reagent (Thermo, United States) following with the manufacturer’s protocol. The RNA was reverse transcribed into cDNA using PrimeScript RT Master Mix Kit or miR-XTM First-Strand Synthesis kit (TaKaRa, Japan). The polymerase chain reaction (PCR) was performed on CFX96 Real-Time PCR System (Bio-Rad, United States) using SYBR Green (Bio-Rad, United States). Fold changes were tested using the 2^–ΔΔCT^ method (Pilli et al., 2017). The data were normalized by GAPDH or U6. The primers were shown as follows: MCP-1 (mouse): forward 5′-CTTCTGGGCCTGCTGTTCA-3′; reverse 5′-CCAGCCTACTCATTGGGATCA-3′; TNF-α (mouse): forward 5′-CATCTTCTCAAAATTCGAGTGACAA-3′; reverse 5′-TGGGAGTAGACAAGGTACAACCC-3′; IL-1β (mouse): forward 5′-TGGGAGTAGACAAGGTACAACCC-3′; reverse 5′-AAGGTCCACGGGAAAGACAC-3′; GAPDH (mouse): forward 5′-GCATGGCCTTCCGTGTTC-3′; reverse 5′-GATGTCATCATACTTGGCAGGTTT-3′; iNOS (mouse): forward 5′-CAGATCGAGCCCTGGAAGAC-3′; reverse 5′-CTGGTCCATGCAGACAACCT-3′; U6 (mouse): forward 5′-GCTCGCTTCGGCAGCACAT-3′; reverse 5′-ATGGAACGCTTCACGAAT-3′; CD86 (mouse): forward 5′-ACTTGAACAACCAGACTCCTGT-3′; reverse 5′-AATAAGCTTGCGTCTCCACG-3′; mmu-miR-21b: forward 5′-CGCGCGTAGTTTATCAGACTGAT-3′; reverse 5′-AGTGCAGGGTCCGAGGTATT-3′; mmu-miR-92b-5p: forward 5′-GGGACGGGACGTGGTGC-3′; reverse 5′-AGTGCAGGGTCCGAGGTATT-3′; mmu-let-7i-5p: forward 5′-CGCGCGTGAGGTAGTAGTTTGT-3′; reverse 5′-AGTGCAGGGTCCGAGGTATT-3′; mmu-miR-374b-5p: forward 5′-GCGCGATATAATACAACCTGC-3′; reverse 5′-AGTGCAGGGTCCGAGGTATT-3′; mmu-miR-676-3p: forward 5′-GCGCCGTCCTGAGGTTGT-3′; reverse 5′-AGTGCAGGGTCCGAGGTATT-3′.

### Periodic Acid–Schiff Staining

The mouse kidney was removed, fixed with 10% formalin for 24 h at 4°C, and then embedded in paraffin. Sections were cut at 5 μm and incubated with 0.5% periodic acid solution for 15 min, followed by stained with Schiff’s reagent for 30 min. After washing by tap water for 10 min, the sections were observed using a light microscope (Avallone et al., 2017).

### Immunohistochemical Analysis

Sections were incubated with primary antibody against CD86 (1:1,000, Abcam) at 4°C overnight, followed by incubated with related secondary antibody (30–40 μL, Abcam) for 30 min at room temperature. The images were captured under a light microscope.

### Cell Culture and Treatment

The mouse TKPTS TEC line, HEK293T cell line, and the mouse RAW264.7 macrophage cell line were obtained from Shanghai Zhong Qiao Xin Zhou Biotechnology (Shanghai, China) and maintained in DMEM/F-12 (Gibco, United States) supplemented with 10% FBS (Gibco, United States) and 1% penicillin-streptomycin (Thermo, United States). The cells were maintained for 24 or 48 h in glucose-free and serum-free culture medium under hypoxic condition (1% O_2_, 5% CO_2_) to stimulate the ischemia *in vitro*. Control cells were cultured under normoxic conditions.

### Fluorescence-Activated Cell Sorting

Isolation of mouse kidney macrophages was as described in this section. Subsequently, antibodies, PE-CD86 (BD Biosciences, Franklin Lakes, NJ, United States) and its isotype-matched negative control antibodies, were added to the cell suspension. After 15 min of incubation in the dark, the cells were washed with PBS and subjected to fluorescence-activated cell sorting (FACS). FACS was performed on a FACSAria and analyzed with FACSDiva4.1 (BD Biosciences).

### Exosome Isolation and Identification

Tubular epithelial cells were maintained in the DMEM medium for exosome isolation with 10% exosome-depleted FBS (System Biosciences Inc., China) for 24 h. The supernatant was collected and centrifuged at 300 × *g* for 15 min, 2,500 × *g* for 30 min, and 10,000 × *g* for 30 min at 4°C. After filtering with a 0.22-mm filter (Millipore, United States), the supernatant was further ultracentrifuged at 100,000 × *g* for 2 h at 4°C, followed by washing with PBS and ultracentrifuged at 100,000 × *g* for 1 h. Then, the pellets were resuspended in 200 μL PBS for further study. For kidney exosome isolation, the kidney cortex tissue was removed, cut, and digested with collagenase and trypsin (Gibco, United States) for 2 h at 37°C. The sample was then collected and subjected to differential centrifugation as described above.

Transmission electron microscope (JEM-2100F, Japan) was used to observe the double-layer capsule ultrastructure of exosomes as the previous study (Rikkert et al., 2019). TECs were stained with the PKH67 dye (Beyotime, China) for 1 h at room temperature. After that, the labeled exosomes were isolated according to the above method. After incubation with macrophages for 48 h, labeled exosomes were visualized by a confocal microscope (FV1000, Olympus, Japan) using a 488-nm laser.

### Exosomes Treatment

Macrophages were incubated with 15 or 30 ng/mL TEC-derived exosomes (in 50 μL PBS) for 48 h. Besides, exosomes (30 ng/mL in 50 μL PBS) derived from inhibitor or control transfected TECs before hypoxia stimulation were added into the macrophage culture system. After 48 h, the macrophages were collected for further study.

### MicroRNA Sequence

The renal exosomes isolated from the mice of sham, day 1 and day 3 group (*n* = 3), were lyzed. The total RNAs were extracted using the miRNeasy Micro Kit (QIAGEN, Germany). Then, we prepared the libraries using TruSeq rapid SBS kit (Illumina, United States) and performed the miRNA profile on Illumina HiSeq 2500 (Illumina, United States) according to the manufacturer’s protocol. The data were analyzed through fold change and *p*-value.

### Cell Transfection

For *in vitro* transfection, 50 nM of mimic control, miR-374b-5p mimic, and miR-374b-5p inhibitor (Synthgene, Nanjing, China) were transfected into cells using Lipofectamine 2000 (Invitrogen, United States), following the manufacturer’s instructions. SOCS1 OE and SOCS1 KD were achieved by transfection with related plasmids (Synthgene, China) into macrophages.

### Western Blot

The exosomes and cells were collected to extract proteins using the RIPA lysis buffer (Beyotime, China) with protease inhibitors (Roche, China). The total proteins were measured by the BCA assay kit (Thermo, United States), and then equal protein (30 μg/lane) was loaded and separated using 10% sodium dodecyl sulfate–polyacrylamide gel electrophoresis. After transferring onto the polyvinylidene fluoride membrane (Millipore, Bedford, MA, United States), the membranes were blocked with 5% skim milk for 2 h at room temperature. Then the membranes were incubated with primary antibodies against CD63 (1:1,000, Abcam), CD9 (1:1,000, Abcam), Alix (1:1,000, Abcam), and SOCS1 (1:2,000, Abcam) at 4°C overnight, followed by incubation with secondary antibody (1:5,000, Abcam) for 2 h. Finally, the blots were detected by using a chemiluminescent detection system (Bio-Rad, United States). GADPH (1:5,000, Abcam) was used as control.

### Immunofluorescence

After different treatments, cells were collected and then fixed with 4% formaldehyde for 15 min at room temperature. After permeabilization with 0.5% Triton X-100 for 15 min, the cells were blocked with 5% goat serum for 1 h, followed by incubating with the antibody against CD86 (1:500, Abcam) overnight at 4°C. The cells were then incubated with the secondary antibody (1:2,000, Abcam) for 2 h at room temperature. DAPI was used to stain cell nuclei according to the manufacturer’s protocol. After immunostaining, samples were observed under a fluorescence microscope.

### Luciferase Reporter Assay

The WT and MUT *Socs1* reporter were obtained from Synthgene (Nanjing, China). These plasmids were cotransfected with miR-374b-5p mimic, inhibitor, or control into HEK293T cells (10^6^ cells per well in six-well plate) using Lipofectamine 2000 (Synthgene, China) according to the manufacturer’s protocol. After 48 h, the luciferase activity was detected and normalized by Renilla luminescence using the kit (Promega, United States) followed with the manufacturer’s protocol.

### Statistical Analysis

All data are shown as means ± SD of three independent experiments. Two-tailed Student *t*-test was used to make comparisons between two groups. One-way analysis of variance was used to make comparisons between multiple groups. *p* < 0.05 was considered statistically significant.

## Results

### M1 Macrophage Was Activated in the Renal Ischemia/Reperfusion-Induced AKI Model

**FIGURE 1:**
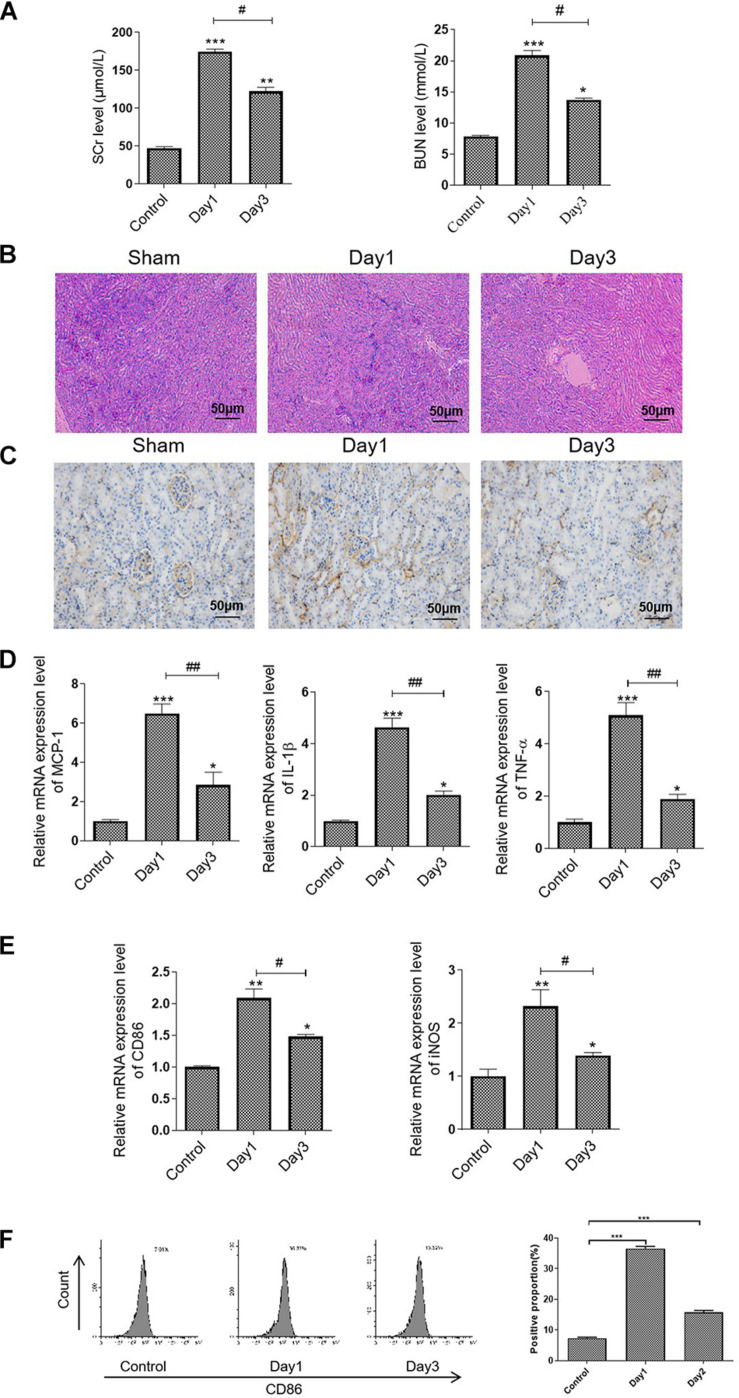
M1 macrophage was activated in the RIR-injured mice model. The mice were grouped and subjected to RIR operation. The serum and kidneys were separated on days 1 and 3 after the operation. **(A)** Serum SCr and BUN levels in the RIR-injured mice. **(B)** Representative periodic acid–Schiff (PAS) staining images of kidneys form RIR-injured or sham mice. Bar = 50 μm. **(C)** Representative IHC images of CD86 in the kidneys from RIR-injured mice were performed. Bar = 50 μm. **(D,E)** The mRNA expression level of inflammatory factors, including MCP-1, TNF-α, IL-1β, CD86, and iNOS in the RIR-injured kidney was assessed by qRT-PCR. **(F)** Expression of CD86 in macrophages was assessed by flow cytometry. Data presented as means ± SD for groups of three mice. *p < 0.05, **p < 0.01, ***p < 0.001 vs. the sham group. ^#^*p* < 0.05, ^##^*p* < 0.01 vs. the day 1 group.

In this study, we first established an RIRI mice model and determined the state of renal damage at different time points. We observed a higher level of SCr and BUN in the mice subjected to bilateral RIR operation on day 1, whereas it was decreased on day 3 (Figure 1A). Further, TEC injury and protein casts were presented in the injured kidneys on day 1, preceding a remission on day 3, related to an increase in CD86-positive macrophages infiltration (Figures 1B,C). The quantitative real-time (qRT) PCR analysis results showed that RIR operation caused a sixfold increase in MCP-1, 5.5-fold increase in TNF-α, and a 4.5-fold increase in IL-1β compared to the sham group on day 1. However, levels of these inflammatory cytokines were markedly decreased on day 3 (Figure 1D). Consistently, the mRNA expression levels of CD86 and iNOS, which were the biomarkers of M1 macrophages, followed a similar pattern, indicating that the M1 macrophages activation was associated with the state of kidney injury (Figure 1E). Furthermore, macrophage polarization was evaluated by flow cytometry for cell surface expression of M1 marker CD86 (Figure 1F).

### miR-374b-5p Increased in Kidney Exosomes From the RIRI Model

**FIGURE 2:**
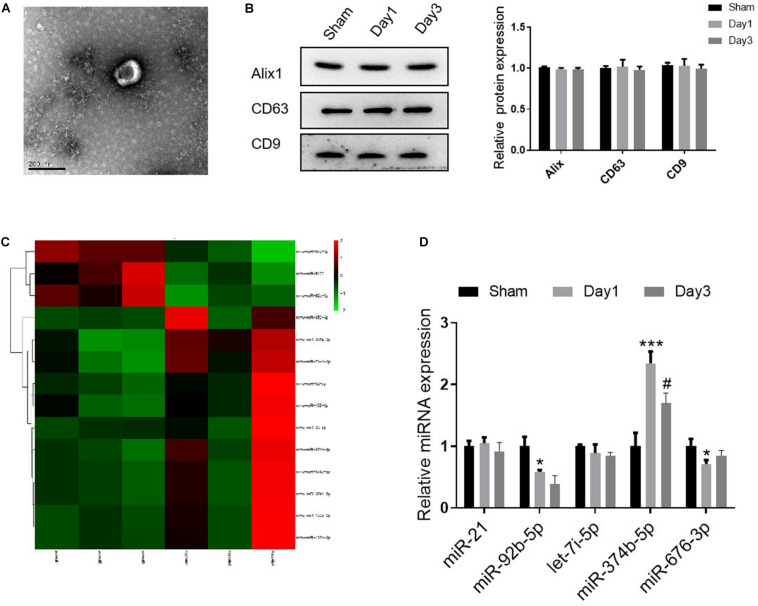
miR-374b-5p increased in kidney exosomes from the RIR-injured model. The kidneys were isolated on days 1 and 3 after RIR operation, and the exosomes were isolated. **(A)** Representative TEM micrograph of exosomes derived from the kidneys. Bar = 200 nm. **(B)** Western blot analysis of Alix, CD63, and CD9 in the kidney released exosomes. **(C)** Heatmap of miRNA profile of kidney exosomes (day 1 group vs. sham group). **(D)** The expression changes of miR-21, miR-92b-5p, let-7i-5p, miR-374b-5p, and miR-676-3p in renal exosomes were measured by qRT-PCR. Data presented as means ± SD for three independent experiments. *p < 0.05, ***p < 0.001 vs. the sham group. ^#^*p* < 0.05 vs. the day 1 group.

Several studies have reported that the transmitting of exosomal miRNA could initiate the inflammatory response to regulate multiple diseases (Simeoli et al., 2017; Pan et al., 2019). For this purpose, we then isolated and characterized the kidney exosomes derived from sham or RIR injured mice (on days 1 and 3 after the operation). Using TEM, we observed that the diameter distribution of kidney exosomes was around 150 nm, consistent with the previous study (Chen H. H. et al., 2014) (Figure 2A). Besides, the Western blot results showed that three exosome markers, including Alix, CD63, and CD9, were expressed in these exosomes (Figure 2B). Next, to explore the miRNA profile of kidney exosomes, we assessed the miRNA signature using the high-throughput sequence. As shown in Figure 2C, compared with the kidney exosomes from sham mice, 14 miRNAs were dramatically changed, with 11 upregulated and three downregulated in the exosomes derived from the kidneys of mice on day 1 after RIR operation. The top five miRNAs with significant upregulation or downregulation relay on the fold change were confirmed by qRT-PCR. Among them, miR-374b-5p was markedly increased, as evidenced by a 2.4-fold incline in the kidney exosomes from day 1 group compared to that in the sham group, whereas its level was decreased in the kidney exosomes from day 3 (Figure 2D). Therefore, miR-374b-5p was selected for further study.

### Inhibiting of miR-374b-5p Alleviated the Renal Injury and Suppressed M1 Macrophage Activation *in vivo*

**FIGURE 3:**
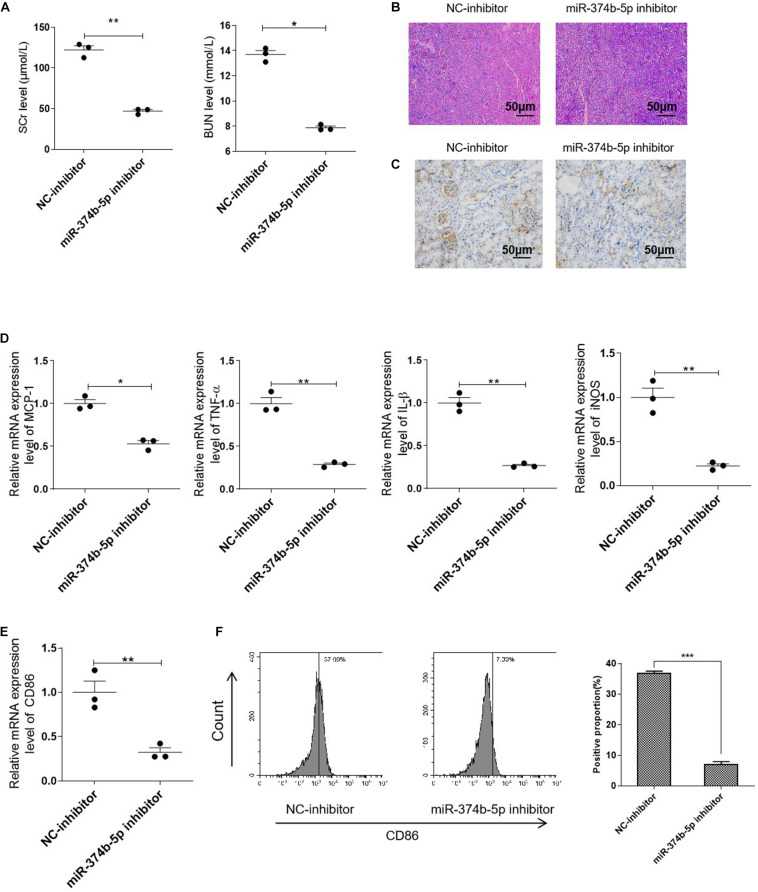
Inhibiting miR-374b-5p alleviated the renal injury and suppressed M1 macrophage activation. The mice were transfected with miR-374b-5p inhibitor and associated control for 48 h before RIR operation. One day after RIR operation, the serum and kidneys were separated. **(A)** Serum levels of SCr and BUN in RIR-injured mice with miR-374b-5p inhibitor or NC-inhibitor transfection were determined. **(B)** Representative PAS staining images of kidneys in mice with or without miR-374b-5p inhibitor treatment. Bar = 50 μm. **(C)** Representative IHC staining images of CD86 from RIR-injured mice after miR-374b-5p inhibitor administration. Bar = 50 μm. **(D,E)** qRT-PCR analysis of MCP-1, TNF-α, IL-1β, CD86, and iNOS in the kidneys of RIR-injured mice with different treatments. **(F)** The quantitative analysis of CD86 positive cells according to flow cytometry results. Data presented as means ± SD for groups of three mice. *p < 0.05, **p < 0.01, ***p < 0.001 vs. the NC-inhibitor group. NC-inhibitor, miRNA inhibitor control.

In order to identify the role of miR-374b-5p, we transfected the miR-374b-5p inhibitor and associated miRNA control (NC-inhibitor) into the kidneys before subjecting to RIR operation. Of note, compared with the NC-inhibitor group, the expression level of SCr was downregulated significantly from 122.32 ± 8.58 to 47.06 ± 3.43 μmol/L 1 day after RIR operation. The BUN level was also decreased from 13.69 ± 0.55 to 7.87 ± 0.23 mmol/L compared to the NC-inhibitor group (Figure 3A). Moreover, miR-374b-5p inhibitor treatment could alleviate kidney injury and reduce protein casts associated with a decrease in CD86-positive macrophages infiltration (Figures 3B,C). Accordingly, assessment of inflammatory cytokines demonstrated a significant decline in the levels of MCP-1, TNF-α, and IL-1β by almost 50, 75, and 75%, respectively, in the miR-374b-5p inhibitor group upon RIR operation, compared to the NC-inhibitor group with the same operation (Figure 3D). We also found that CD86 and iNOS expression levels were dramatically reduced by 75 and 80%, respectively, in the kidneys of mice pretransfected with miR-374b-5p inhibitor upon RIR operation as compared to the NC-inhibitor group (Figure 3E). Besides, decrease in CD86 expression was measured by FACS in the miR-374b-5p inhibitor group (Figure 3F). Our results suggested that the miR-374b-5p inhibitor’s transfection could attenuate kidney injury and inhibit M1 macrophage activation *in vivo*.

### miR-374b-5p Increased in the TECs and Related Exosomes Under the Hypoxic Condition

**FIGURE 4:**
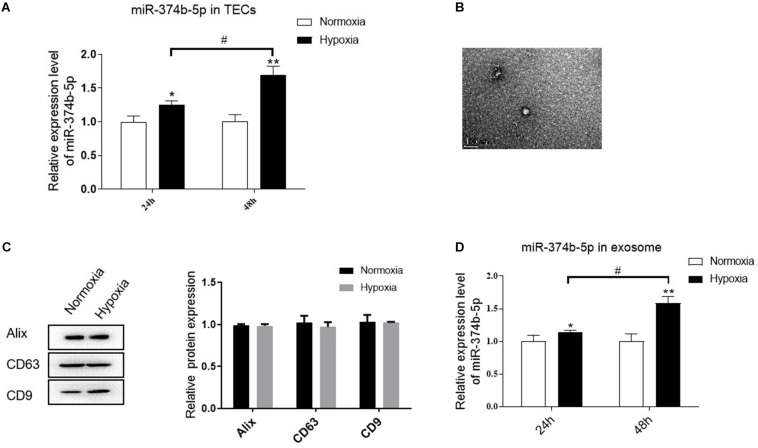
miR-374b-5p increased in the TECs and related exosomes under the hypoxic condition. **(A)** The expression level of miR-374b-5p in the TECs with normoxia or hypoxia exposure for 24 or 48 h. **(B)** Representative TEM image of TEC-derived exosomes. Bar = 200 nm. **(C)** Western blot analysis of Alix, CD63, and CD9 in the exosomes derived from TECs subjected to hypoxic or normoxic conditions for 24 h. **(D)** miR-374b-5p expression in exosomes derived from hypoxic or normoxic TECs at different timepoints (24 or 48 h) was assessed by qRT-PCR. *N* = 3, data presented as means ± SD. *p < 0.05, **p < 0.01 vs. the normoxia group. ^#^*p* < 0.05 vs. the hypoxia (24 h) group.

We then examined whether miR-374b-5p was selectively enriched in the renal exosomes, especially in the TEC-derived exosomes. TECs were exposed to normoxic or hypoxic conditions, and the exosomes were then isolated after 24 or 48 h. Compared to the normoxia group, the level of miR-374b-5p in TECs was increased time-dependently, with a 20% increase at 24 h and 60% upregulation at 48 h (Figure 4A). Moreover, TEM results showed that the size and structure of TEC-derived exosomes were similar to the previous studies (Figure 4B) (Chen H. H. et al., 2014). Western blot analysis indicated that the expression of Alix, CD63, and CD9, three common markers of exosomes, presented in both normoxic and hypoxic TEC-derived exosomes (Figure 4C). Next, we measured the expression level of miR-374b-5p in exosomes from TECs. As respected, the level of miR-374b-5p gently increased in exosomes from TECs subjected to hypoxic conditions for 24 h, whereas it was elevated by 1.6-fold when the cells were exposed to hypoxia for 48 h (Figure 4D).

### Exosomal miR-374b-5p Derived From Hypoxia-Induced TECs Promoted M1 Macrophage Activation *in vitro*

**FIGURE 5:**
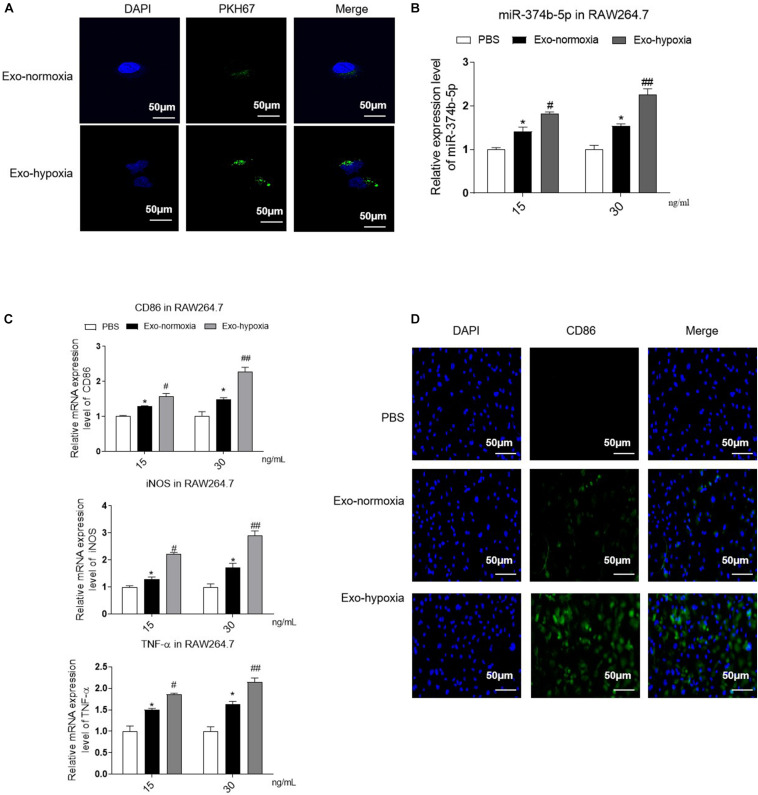
Uptake of exosomes-hypoxia by macrophage promoted M1 macrophage activation. Exosomes were isolated from the TECs, which were exposed to hypoxic or normoxic conditions for 48 h and then were applied to treat naive macrophages at different concentrations (15 or 30 ng/mL) for 48 h. **(A)** PKH67-labeled exosomes uptake by macrophages. Bar = 50 μm. **(B)** The expression level of miR-374b-5p in macrophages was detected by qRT-PCR. **(C)** qRT-PCR analysis of TNF-α, CD86, and iNOS in the macrophages with different treatments. **(D)** Representative images of CD86 in recipient macrophages were detected by IF. Bar = 50 μm. *N* = 3, data presented as means ± SD. *p < 0.05 vs. the PBS group. ^#^*p* < 0.05, ^##^*p* < 0.01 vs. the Exo-normoxia group. Exo-normoxia, exosomes derived from the TECs under the normoxic condition for 48 h. Exo-hypoxia, exosomes derived from the TECs, which were exposed to hypoxia for 48 h.

To evaluate the effect of exosomes from TECs under hypoxic conditions, we treated the naive macrophages with these exosomes for 48 h. As shown in Figure 5A, clear PKH67 green signals were observed inside the macrophages, and the level was higher in the exosomes-hypoxia-treated group than exosomes-normoxia-treated group. Moreover, the qRT-PCR result demonstrated that miR-374b-5p expression was also dose-dependently upregulated in the macrophages after hypoxic TEC-derived exosomes treatment, with 1.82 ± 0.06 at 15 ng/mL and 2.25 ± 0.24 at 30 ng/mL (*p* < 0.05). Of note, when the naive macrophages were treated with exosomes-normoxia, the expression level of miR-374b-5p was also significantly upregulated at both doses, whereas they were lower than the exosomes-hypoxia groups (Figure 5B). This was also consistent with the dramatic production of CD86, iNOS, and TNF-α (Figure 5C). Notably, the immunofluorescence results showed that the macrophages treated with exosomes-hypoxia exhibited a higher level of CD86 than other groups (Figure 5D).

**FIGURE 6:**
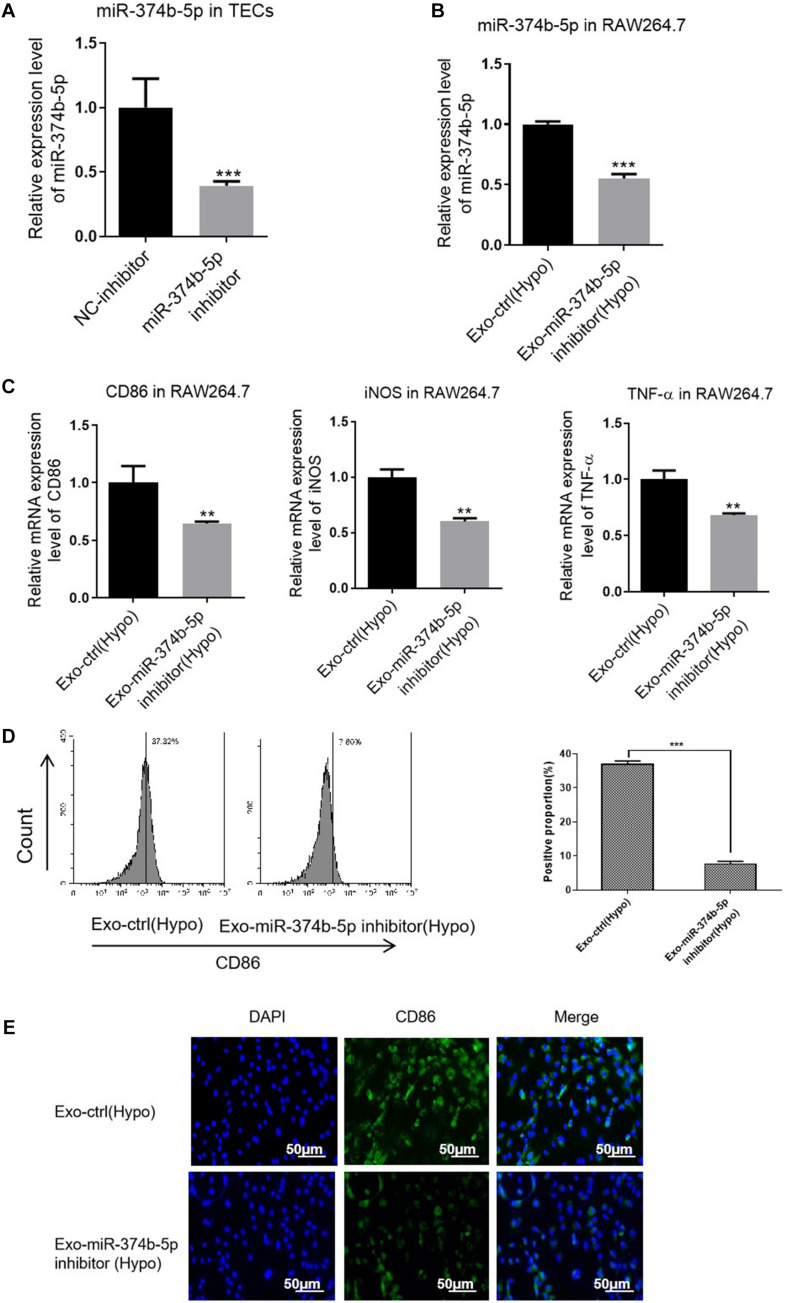
Exosomal miR-374b-5p inhibition repressed the M1 macrophage activation. The TECs were transfected with miR-374b-5p inhibitor and related control (NC-inhibitor), followed by exposed to hypoxic conditions for 48 h. Then the exosomes were isolated and applied to treat the naive macrophages. **(A)** qRT-PCR analysis of miR-374b-5p in the TECs with miR-374b-5p inhibitor or related control transfection. ***p < 0.001 vs. the NC-inhibitor group. **(B)** The expression level of miR-374b-5p in macrophages after different treatments were determined by qRT-PCR. **(C)** The expression of TNF-α, CD86, and iNOS in the macrophages after exosome administration was evaluated by qRT-PCR. **(D)** The proportion of CD86-positive cells was presented. **(E)** Representative images of CD86 in macrophages with different treatments were detected by IF. Bar = 50 μm. Data presented as means ± SD for three independent experiments. **p < 0.01, ***p < 0.001 vs. the Exo-ctrl (Hypo) group. Exo-ctrl (Hypo), exosomes derived from the NC-inhibitor transfected TECs, were then subjected to hypoxic conditions for 48 h. Exo-miR-374b-5p inhibitor (Hypo), exosomes derived from the miR-374b-5p inhibitor transfected TECs, were then subjected to hypoxic conditions for 48 h. NC-inhibitor, miRNA inhibitor control.

Additionally, we transfected miR-374b-5p inhibitor and associated control into TECs. With the transfection of inhibitor, miR-374b-5p expression in the TECs was significantly declined (Figure 6A). The TECs were then maintained under the hypoxic condition for 48 h. After that, the exosomes were isolated and applied to naive macrophages. The results showed that the expression level of miR-374b-5p was lower in the exosome-miR-374b-5p inhibitor (Hypo) group than that in the associated control [exo-ctrl (Hypo)] group (Figure 6B). Consistently, treatment with miR-374b-5p-inhibited exosomes markedly decreased the mRNA level of CD86, iNOS, and TNF-α in macrophages (Figure 6C). Decrease in CD86 expression was measured by FACS in the miR-374b-5p-inhibitor group (Figure 6D). Besides, the CD86 expression, assessed by immunofluorescence assay, followed a similar pattern (Figure 6E).

### Exosomal miR-374b-5p Prompted M1 Macrophage Activation via SOCS1

**FIGURE 7:**
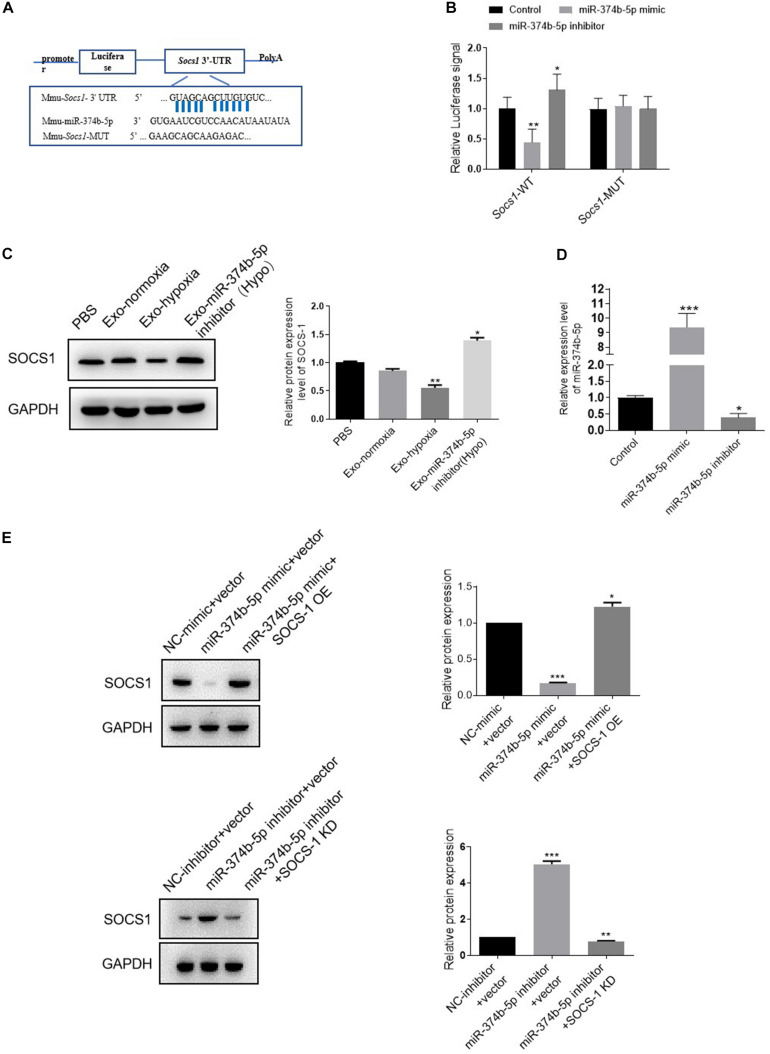
Exosomal miR-374b-5p prompted M1 macrophage activation via directly targeting *Socs1*. **(A)** The predicted binding site between miR-374b-5p and *Socs1* 3’-UTR. **(B)** The activities of luciferase reporters containing wild-type (WT) or mutant (MUT) *Socs1* 3’-UTR were transfected into the HEK293T cells that were then treated with miR-374b-5p mimic, miR-374b-5p inhibitor, or respective control, respectively. *p < 0.05, **p < 0.01 vs. the control group. **(C)** Protein expression of SOCS1 in macrophages treated with exosomes-normoxia, exosomes-hypoxia, exosome-miR-374b-5p inhibitor (Hypo), and PBS treatment. *p < 0.05, **p < 0.01 vs. the PBS group. **(D)** mRNA expression of miR-374b-5p in macrophages after miR-374b-5p mimic, miR-374b-5p inhibitor, or related control transfection. *p < 0.05, ***p < 0.001 vs. the control group. **(E)** Western blot analysis of SOCS1 in macrophages after miR-374b-5p mimic, miR-374b-5p inhibitor, or SOCS1 overexpression vector (SOCS1 OE) or SOCS1 knockdown vector (SOCS1 KD) transfection. *p < 0.05, **p < 0.01, ***p < 0.001 vs. the NC-mimic + vector group or the NC-inhibitor + vector group. Data presented as means ± SD for three independent experiments. Exo-normoxia, exosomes from TECs exposed to normoxia for 48 h. Exo-hypoxia, exosomes from TECs exposed to hypoxic conditions for 48 h. Exo-miR-374b-5p inhibitor (Hypo), exosomes from miR-374b-5p inhibitor transfected TECs prior to hypoxia treatment for 48 h. Control, mimic control + miRNA inhibitor control.

Next, we sought to investigate the underlying mechanism of miR-374b-5p. Based on this purpose, the bioinformatics database^1^ was used to predict the potential targets of miR-374b-5p. As expected, suppressor of cytokine signaling (*Socs1*) was found, and the binding sites between miR-374b-5p and *Socs1* are shown in Figure 7A. Besides, the luciferase reporter assay results also confirmed that miR-374b-5p mimic dramatically declined the fluorescence activity of *Socs1*-WT rather than *Socs1*-MUT, suggesting that *Socs1* was a direct target gene of miR-374b-5p (Figure 7B). We then determined the SOCS1 expression in macrophages treated by exosomes from TECs after different treatments. The results demonstrated that the expression of SOCS1 was concomitantly inhibited in the exosomes-hypoxia group related to the PBS group or the exosomes-normoxia group, whereas miR-374b-5p inhibitor significantly reversed this effect (Figure 7C). Moreover, OE of miR-374b-5p markedly inhibited the expression of SOCS1, as evidenced by an 80% reduction in the miR-374b-5p mimic-treated cells. In contrast, it was diminished by pretransfected with SOCS1 OE vector. However, miR-374b-5p silencing increased the SOCS1 expression compared to the control group, which would be reversed when we pretransfected the SOCS1 KD vector into macrophages (Figures 7D,E). Our results indicated that exosomal miR-374b-5p might activate macrophages by suppressing the expression of SOCS1.

### The Effect of Exosomes Secreted From Hypoxic TECs *in vivo*

**FIGURE 8:**
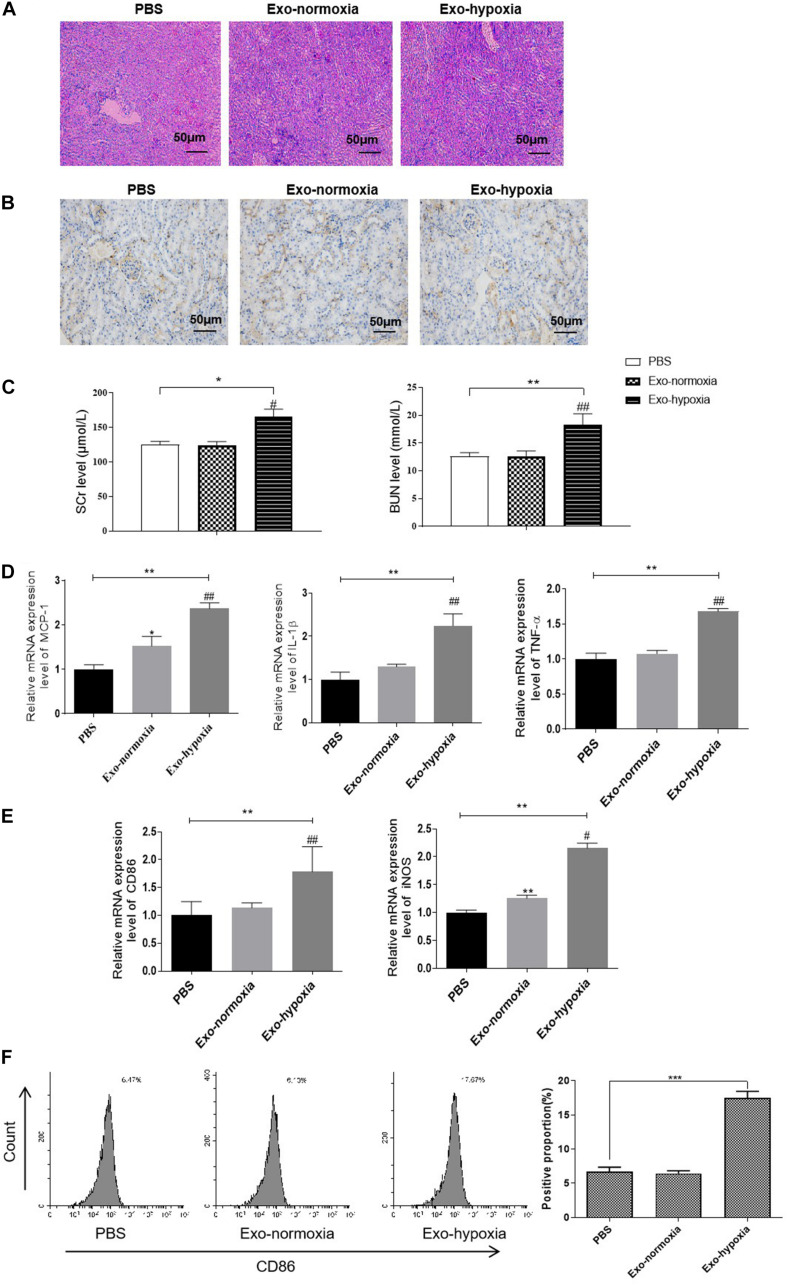
Exosomal miR-374b-5p led to renal inflammation response and M1 macrophage activation. Exosomes were isolated from the TECs, which were exposed to hypoxic or normoxic conditions for 48 h and then were injected into the kidney of mice. One day after the RIR operation, the serum and kidneys were separated. **(A)** The PAS staining images of kidneys after PBS, exosomes-normoxia, or exosomes-hypoxia injection. Bar = 50 μm. **(B)** Representative IHC images of CD86 in the kidneys after exosomes injection. Bar = 50 μm. **(C)** Serum levels of SCr and BUN in RIR-injured mice with exosomes or PBS treatment were performed. **(D,E)** mRNA expression of MCP-1, TNF-α, IL-1β, CD86, and iNOS in the exosomes-injected kidneys were determined by qRT-PCR. **(F)** The proportion of CD86-positive cells was analyzed by FACS. Data presented as means ± SD for groups of three mice. *p < 0.05, **p < 0.01, ***p < 0.001 vs. the PBS group. ^#^*p* < 0.05, ^##^*p* < 0.01 vs. the Exo-normoxia group. Exo-normoxia, exosomes from TECs exposed to normoxic condition for 48 h. Exo-hypoxia, exosomes from TECs exposed to hypoxic conditions for 48 h.

To determine the function of exosomes derived from hypoxic TECs *in vivo*, we then injected these exosomes into the kidney of mice. The mice were then subjected to RIR operation. One day after exosome treatment, a higher degree of renal tubulointerstitial injury was exhibited when injected with the exosomes-hypoxia compared with the control injection with exosomes from normoxic TECs (Exo-normoxia) (Figure 8A). Further, the immunohistochemical analysis demonstrated that exosomes-hypoxia treatment increased the intensity of CD86 in the injured kidneys than that in the other groups (Figure 8B). Besides, the levels of SCr and BUN were markedly upregulated after exosome-hypoxia treatment compared to the exosome-normoxia group, whereas there was no difference between the exosome-normoxia-treated group and the sham group (Figure 8C). The mRNA expression of MCP-1, TNF-α, and IL-1β was also significantly upregulated in the exosomes-hypoxia group (Figure 8D). Accordingly, a higher mRNA level of CD86 and iNOS was observed in the kidneys after exosomes-hypoxia treatment (Figure 8E). Besides, increase in CD86 expression was measured by FACS in the Exo-hypoxia group (Figure 8F). These findings suggest that hypoxic TEC-derived exosomes induced kidney injury via activating macrophages. Generally, we hypothesized whether inhibition of exosomal miR-374b-5p from hypoxic TECs would abolish this effect.

### Exosomal miR-374b-5p Led to Renal Inflammation Response and M1 Macrophage Activation

**FIGURE 9:**
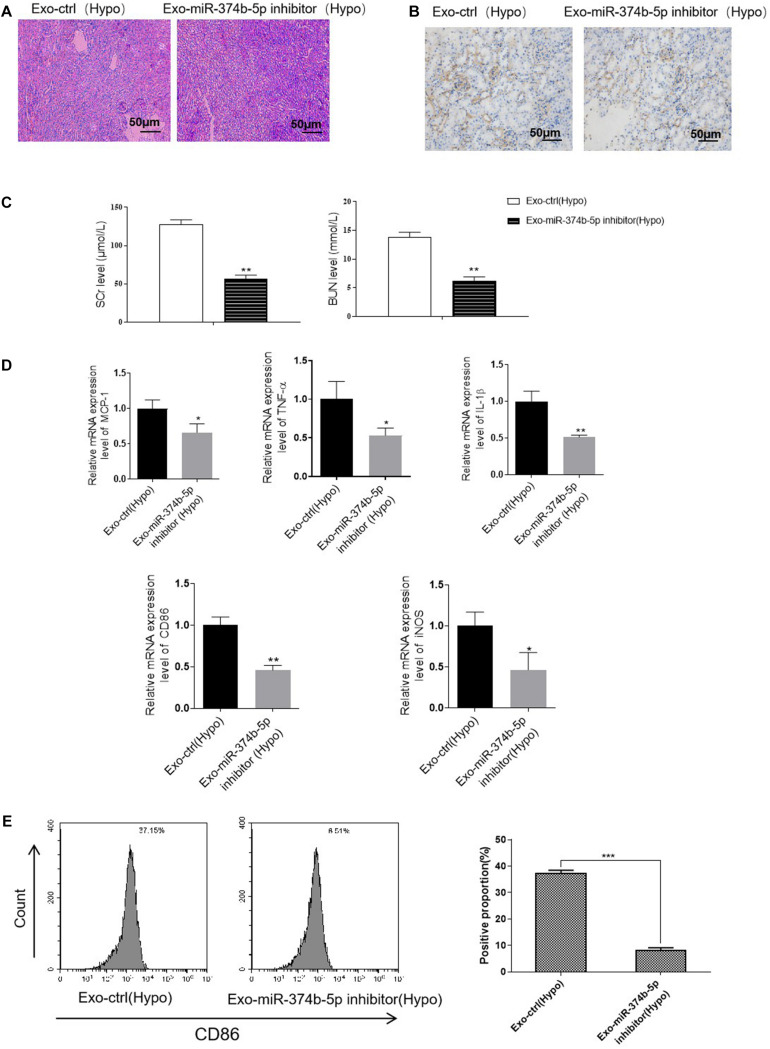
Inhibition of exosomal miR-374b-5p alleviated RIRI in mice. The TECs were transfected with miR-374b-5p inhibitor or related control, followed by exposed to hypoxic conditions for 48 h. Then the exosomes were isolated and injected into the mice. The mice were then subjected to RIRI, and the kidney tissues and serum were collected on day 1 after the operation. **(A)** Representative PAS images of kidneys at 48 h after different treatments. Bar = 50 μm. **(B)** IHC assessed the expression of CD86 in the kidney from mice with different treatments. Bar = 50 μm. **(C)** Serum levels of SCr and BUN in RIR-injured mice with exosomes-ctrl (Hypo) or exosomes-miR-374b-5p inhibitor (Hypo) treatment were performed. **(D)** mRNA expression of MCP-1, TNF-α, IL-1β, CD86, and iNOS in the kidney injected with exosomes-ctrl (Hypo) or exosome-miR-374b-5p inhibitor (Hypo) was detected by qRT-PCR. **(E)** The proportion of CD86-positive cells was determined by flow cytometry. Data presented as means ± SD for groups of three mice. *p < 0.05, **p < 0.01 vs. the Exo-ctrl (Hypo) group. Exo-ctrl (Hypo), exosomes derived from the hypoxic TECs after NC-inhibitor transfection. Exo-miR-374b-5p inhibitor (Hypo), exosomes derived from the hypoxic TECs after miR-374b-5p inhibitor transfection.

To confirm our hypothesis, we treated the hypoxic TECs with miR-374b-5p inhibitor or related control (inhibitor-NC) and injected their exosomes [Exo-miR-374b-5p inhibitor (Hypo) or Exo-ctrl (Hypo)] into mice kidney. The mice were then subjected to RIR surgery. One day after RIR operation, we observed that exosomes-miR-374b-5p inhibitor (Hypo) could alleviate the RIR injury, with lower infiltration of CD86-positive macrophage than that in the related control [Exo-ctrl (Hypo)] group (Figures 9A,B). Besides, SCr and BUN levels were markedly downregulated after treatment with the miR-374b-5p-inhibited exosomes derived from hypoxic TECs (Figure 9C). Furthermore, as shown in Figure 9D, compared with the exosomes-ctrl (Hypo) group, the expression of inflammatory cytokines (MCP-1, TNF-α, and IL-1β) in the kidney was markedly reduced after exosomes-miR-374b-5p inhibitor (Hypo) treatment. Consistently, a reduction of CD86 and iNOS expression in exosomes-miR-374b-5p inhibitor (Hypo) recipients was also detected by qRT-PCR. Besides, decrease in CD86 expression was measured by FACS in the miR-374b-5p-inhibitor group (Figure 9E). These results suggested that hypoxic TECs contributed to RIRI via transferring exosomal miR-374b-5p into naive macrophages to activate their activation.

## Discussion

The mechanism by which the macrophages are instructed to alter their phenotype and functional repertoire during RIRI is still unknown. The present study demonstrated that hypoxic TECs significantly activated M1 macrophage during RIRI formation. This harmful effect was achieved by transferring exosomal miR-374b-5p to macrophages, which further suppressed the expression of SOCS1. In addition, hypoxic exosomes treatment significantly induced the production of the inflammatory cytokines and worsened the kidney injury *in vivo*. Our results might provide a potential therapeutic strategy for RIRI treatment.

Recent studies have shown that hypoxia acted as an essential player in renal failure, which not only damages the TECs but also initiates the inflammatory response (Tanaka et al., 2014). Such inefficiency in oxygen supply during ischemia predisposes the kidney to damage. This study successfully established an RIRI mice model, as evidenced by increased renal dysfunction markers and apparent tubular damage. Moreover, the expression of inflammatory factors, including MCP-1, IL-1β, and TNF-α, increased dramatically, which was consistent with previous studies (Moayeri et al., 2003; Saenz-Morales et al., 2010). After RIR injury, upregulated expression of CD86 and iNOS, the biomarkers of M1 macrophage polarization, was observed on day 1 after RIR operation. These results indicated that macrophage recruitment early after RIRI had a proinflammatory phenotype—M1 macrophage.

We then revealed the miRNA signature of injured kidneys exosomes using a high-throughput sequence. Among them, miR-374b-5p was selectively packaged into the exosomes after RIR operation. Subsequently, when we transfected the mice with miR-374b-5p before RIR operation, the proinflammatory cytokines were decreased, and M1 polarization was inhibited. It had been proven that TECs could trigger M1 polarization under hypoxic conditions *in vitro* or subjected to RIR operation *in vivo*. We thus considered whether exosomal miR-374b-5p acted as the bridge between TECs and macrophages. Our *in vitro* studies further confirmed that miR-374b-5p was expressed in TECs and also enriched in associated exosomes when subjected to hypoxic conditions. Therefore, we believed that exosomal miR-374b-5p might represent a new messenger for cell–cell communication during kidney injury.

To identify the role of exosomal miR-374b-5p, we isolated the exosomes from hypoxic TECs and applied them to macrophages *in vitro* or kidneys *in vivo*. We found that miR-374b-5p-enriched exosomes could be taken up by macrophages and consequently activate these recipients. When we injected the exosomes derived from hypoxic TECs into mice, severe kidney damage and upregulated proinflammatory factors have been observed. By contrast, exosomes from miR-374b-5p inhibitor-transfected TECs with hypoxia stimulation did not trigger M1 macrophage activation and promote kidney damage *in vitro* and *in vivo*. Hence, it suggested that exosomal miR-374b-5p might be an essential mediator for RIRI formation.

Furthermore, with the bioinformatics analysis, we found that miR-374b-5p promoted macrophage activation via suppression of Socs1. Downregulating SOCS1 expression was known to promote macrophages switching to the M1 phenotype (Liang et al., 2017). Also, *Socs1* could be a potential target of multiple miRNAs in various diseases (Nie et al., 2016; Lv et al., 2020). In the current study, miR-374b-5p-enriched exosomes inhibited SOCS1 expression, which presented a similar result with the miR-374b-5p treatment. Inversely, silencing miR-374b-5p increased the level of SOCS1 in macrophages. We thus believed that SOCS1 could be involved in macrophage switch and regulate the inflammation during RIRI progression.

## Conclusion

In summary, our findings supported that exosomal miR-374b-5p plays a crucial role in the inflammatory response during RIRI, including M1 polarization and the production of inflammatory cytokines. Thus, the downregulation of miR-374b-5p exhibited a protective effect against RIRI, which might consider being a promising molecular therapeutic target for RIRI treatment.

## Data Availability Statement

The original contributions presented in the study are included in the article/supplementary material. Further inquiries can be directed to the corresponding author/s.

## Ethics Statement

The animal study was reviewed and approved by the ethic committee of the First Affiliated Hospital of Xi’an Jiaotong University.

## Author Contributions

CGD and JZ designed and analyzed the experiments and were major contributors to the manuscript. The remaining authors conducted separate experiments. All authors read and approved the final manuscript.

## Conflict of Interest

The authors declare that the research was conducted in the absence of any commercial or financial relationships that could be construed as a potential conflict of interest.

## Footnotes

1 www.targetscan.org

## Abbreviations

AKI: acute kidney injury
BUN: blood urea nitrogen
ctrl: inhibitor control
DAPI: 4,6-diamidino-2-phenylindole
DMEM: Dulbecco modified Eagle medium
Exo: exosome
FBS: fetal bovine serum
Hypo: hypoxia
IL-1 β: interleukin-1 β
iNOS: inducible nitric oxide synthase
i.p.: intraperitoneal injection
IRI: ischemia and reperfusion injury
KD: knockdown
MCP-1: monocyte chemoattractant protein-1
miRNAs: microRNAs
MUT: mutant
OE: overexpression
PAS: periodic acid-Schiff
RIRI: renal ischemia/reperfusion injury
SCr: serum creatinine
SOCS1: suppressor of cytokine signaling-1
TECs: tubular epithelial cells
TEM: transmission electron microscope
TNF- α: tumor necrosis factor- α
WT: wild-type.
